# Comparison of the Optical Quality between Small Incision Lenticule Extraction and Femtosecond Laser LASIK

**DOI:** 10.1155/2016/2507973

**Published:** 2016-11-10

**Authors:** Ying Jin, Yan Wang, Lulu Xu, Tong Zuo, Hua Li, Rui Dou, Jiamei Zhang

**Affiliations:** Tianjin Eye Hospital, Tianjin Eye Institute, Tianjin Key Lab of Ophthalmology and Visual Science, Tianjin Medical University and Nankai University, Tianjin 300020, China

## Abstract

*Purpose.* To investigate the visual quality after SMILE and Femto-LASIK.* Methods.* About 123 eyes from 63 patients were enrolled in this study. The parameters were measured preoperatively and 1 week, 1 month, and 3 months postoperatively using Sirius System.* Results.* The MTF curve increase slightly from low to high frequency at 3 mm and 6 mm pupil diameter after SMILE surgery (*P* < 0.05) during the follow-up time comparing with the preoperative values. And the SR has a significant increase at various pupil diameters as well (*P* < 0.05). However, there was little increase for MTF at intermediate to high frequency at 3 mm pupil diameter after Femto-LASIK procedure (*P* < 0.05). And the SR had only significant increase at 3 mm pupil diameter. Between SMILE and Femto-LASIK, there was no statistic difference both in MTF and SR (*P* > 0.05) at 3 mm pupil diameter at vertical and horizontal meridian. However, significant difference was found in MTF at low to mediate frequency at 6 mm pupil diameter at vertical meridian at 1 week postoperatively (*P* < 0.05).* Conclusions.* Both SMILE and Femto-LASIK show a great improvement in optical quality at small diameter. It was found that SMILE shows better optical quality than Femto-LASIK at larger pupil diameter, which might be good for the night vision.

## 1. Introduction

Small incision lenticule extraction (SMILE), without lifting a flap, has been proposed as an alternative to conventional LASIK for the refractive correction. Interestingly, the recovery of the visual acuity after this novel technique has been found to be slightly slower than that after other techniques in the early postoperative period [1, 2]. Whether the optical quality of the early stage after surgery is affected by this phenomenon is not clear.

Modulation transfer function (MTF) and point spread function (PSF) are two parameters to evaluate optical quality. MTF indicates the ability of cornea to transfer various levels of detail from the object to the image. As a function of spatial frequency, its units are the ratio of image contrast over the object contrast. Physical optics theory demonstrated that any object is composed of an infinite array of point sources, each with its respective intensity, position, and color. Convolution operation gives each point of the PSF shape. Thus, PSF is the image that an optical system forms of a point source which is the fundamental object and forms for any complex object. It takes into account diffraction effect and is usually evaluated as Strehl ratio (SR). Therefore, this study was conducted to evaluate the optical quality after SMILE procedure using these two objective parameters, MTF and SR. And the results were also compared with those after Femto-LASIK procedure in order to evaluate them objectively and comprehensively.

## 2. Participants and Methods

In this prospective clinical comparative study, 63 eyes of 32 patients who underwent SMILE and 61 eyes of 31 patients who underwent Femto-LASIK for the correction of myopia and myopic astigmatism were included. Informed consent was obtained from each patient and the tenets of the Declaration of Helsinki were followed. This study was approved by the Institutional Review Board and Ethics Committee of Tianjin Eye Hospital, Tianjin, Chinese. The treatment eligibility criteria were identical for both groups: spherical myopia up to −10 diopters (D) and myopic astigmatism up to −4D cylinders. Other criteria were a minimum age of 21 years, corrected distance visual acuity (CDVA) ≥ 0.8 (20/25) and no other ocular diseases except myopia and astigmatism. The central corneal thickness had to be more than 480 *μ*m, and the calculated residual stromal bed after treatment should more than 280 *μ*m. The patients were matched for similar age, refractive error, and other preoperative parameters as shown later in Table 1. A regular corneal topographic shape was verified by SIRIUS topography and values at 3 mm and 6 mm pupil diameter of cornea were included in consideration of the pupil diameters' effect on MTF and SR before the procedures. All patients had a bilateral simultaneous procedure. Our routine follow-up times were 1 week, 1 month, and 3 months.

### 2.1. Examination Instruments

3D Sirius-Complete Anterior Segment Imaging System (Italy CSO, SIRIUS, software version: phoenis 1.2), which includes Scheimpflug tomographic mapping of the cornea for shape, was used to measure the MTF curves at various spatial frequencies (10, 20, 30, 40, 50, and 60 cpd) and SR values at 3 mm and 6 mm pupil diameter of cornea. All examinations were operated by the same expert technician. The examination was conducted immediately after blink eyes under natural light. The patients were told to stare at the blue fixation light of the corneal topography. Three eligible measurements were taken and the subjects were excluded if the three scans were of poor quality. The one of highest quality was chosen for analysis. The repeatability and reliability of SIRIUS for measuring segment parameters have been demonstrated [3–5].

### 2.2. Surgical Techniques

SMILE and Femto-LASIK procedure were performed under topical anesthesia (Benoxil, Santen, Inc., Osaka, Japan) and all eyes were performed by the same surgeon (Y. W.).

### 2.3. ReLEx SMILE Procedure

In ReLEx SMILE procedure, a femtosecond laser system (Carl Zeiss Meditec AG VisuMax) with a repetition rate of 500 kHz was used to perform the whole surgical. The cap thickness set at 110 *μ*m and the diameter was set 7.0~7.5 mm. The lenticule size was 6.2 ± 0.2 mm (range 6.0~6.5 mm) with no transition zone for spherical aberration and an 0.1 mm transition zone for astigmatism correction. A side-cut incision angle was set 90° and the side-cut incision was set at 2~4 mm. the ablation energy was 110~175 nJ. The ablation order was as follows: the posterior surface of the lenticule; the anterior surface of the lenticule; the side cut and the side-cut incision. The lenticule was then separated bluntly and removed with a forceps through the side-cut incision.

### 2.4. Femto-LASIK Procedure

The same femtosecond laser system was used in the Femto-LASIK group. Eyes had flap creation performed with a 110 *μ*m flap thickness and programmed flap diameters of 7.9~8.0 mm. standard 90° hinges and 90° side-cut angles. Stromal tissue ablation was performed with excimer laser system (Allegretto; WaveLight Laser Technologie AG, Erlangen, Germany) whose repetition frequency was 400 kHz. The pulse energy was 150 nJ and the ablation diameter of the Gaussian spot profile was 1.0 mm. Eyes had ablations using an optical zone diameter of 6.0~6.5 mm surrounded by a transition zone of 1.0~1.2 mm.

Both in the ReLEx SMILE group and in Femto-LASIK group, topical ofloxacin 0.3% (Tarivid; Santen, Inc., Osaka, Japan) was applied 4 times daily for 2 days postoperatively. 0.1% fluorometholone (Flumetholon; Santen, Inc., Osaka, Japan) was applied 4 times per day for 2 weeks and then tapered over 2 months.

### 2.5. Statistical Analysis

All statistical analyses were performed by SPSS (20.0 USA). Repeat one-way analysis of variance (ANOVA) was used for the analysis of the time course of changes after surgery. The normality of all data samples was first checked by the Kolmogorov–Smirnov test. The Wilcoxon signed rank test was used for statistical analysis to compare the uncorrected distance visual acuity (UDVA) and manifest spherical equivalent between the two groups. The difference change of MTF values, Strehl ratio, and RMS values between SMILE and Femto-LASIK groups were tested by repeat one-way analysis of variance (ANOVA). The results are expressed as mean ± SD.* P* value less than 0.05 was considered statistically significant.

## 3. Results

The preoperative characteristics of these two groups were shown in Table 1. There was no significant difference in the preoperative parameters between the two groups.

### 3.1. Visual Acuity and Refraction

The preoperative Log MAR UDVA was 1.10 ± 0.23 (0.50 to 1.54) in SMILE group and 1.08 ± 0.29 (0.50 to 1.52) in Femto-LASIK group. There was no significant difference in Log MAR UDVA between two groups. Three months postoperatively, the Log MAR UDVA was −0.17 ± 0.10 (range 0.00 to −0.30) in SMILE group and in Femto-LASIK group the Log MAR UDVA was −0.14 ± 0.10 (range 0.10 to −0.30). The manifest spherical equivalent in SMILE group was −0.05 ± 0.26 (range: −0.50 to +1.25) and −0.15 ± 0.26 (range: −0.75 to +1.25) for Femto-LASIK group. Wilcoxon signed rank test showed that there were no significant differences in terms of Log MAR UDVA (*P* = 0.42) and manifest spherical equivalent (*P* = 0.58).

### 3.2. Changes of MTF after SMILE

**Figure 1:**
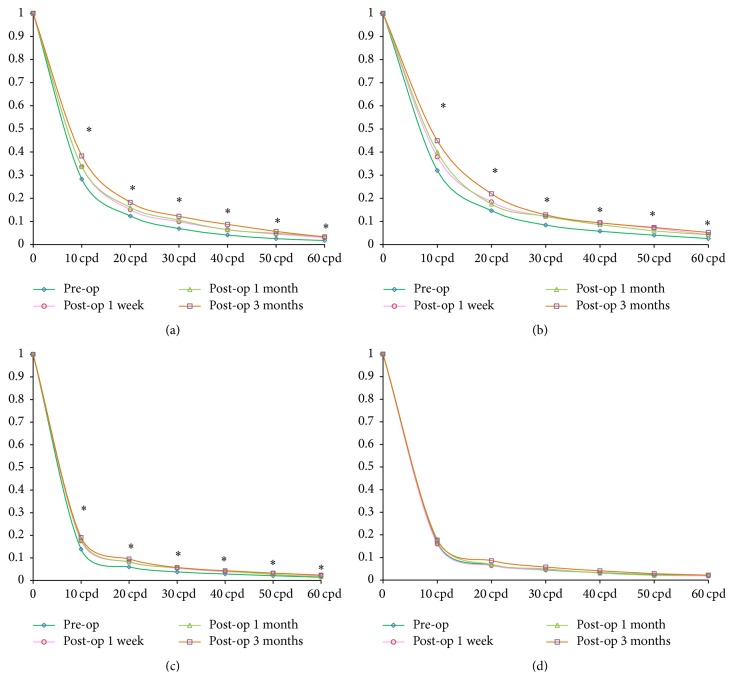
The changes of MTF values before and 1 week, 1 month, and 3 months after SMILE procedure. (a) The change of MTF curve at vertical meridian at 3 mm pupil diameters; (b) the change of MTF curve at horizontal meridian at 3 mm pupil diameters; (c) the change of MTF curve at vertical meridian at 6 mm pupil diameters; (d) the change of MTF curve at horizontal meridian at 6 mm pupil diameters. ^*∗*^Significant difference among different follow-up times.

The MTF curve of the corneal surface at vertical and horizontal meridian increase significantly from low to high frequency at 3 mm pupil diameter after SMILE procedure (*P* < 0.05 for all) during the follow-up time (Figures 1(a) and 1(b)). However, the MTF values only showed significant increase at vertical meridian from low to high frequency at 6 mm pupil diameter before and after SMILE procedure (*P* < 0.05 for all) (Figures 1(c) and 1(d)).

### 3.3. Changes of MTF Values after Femto-LASIK

**Figure 2:**
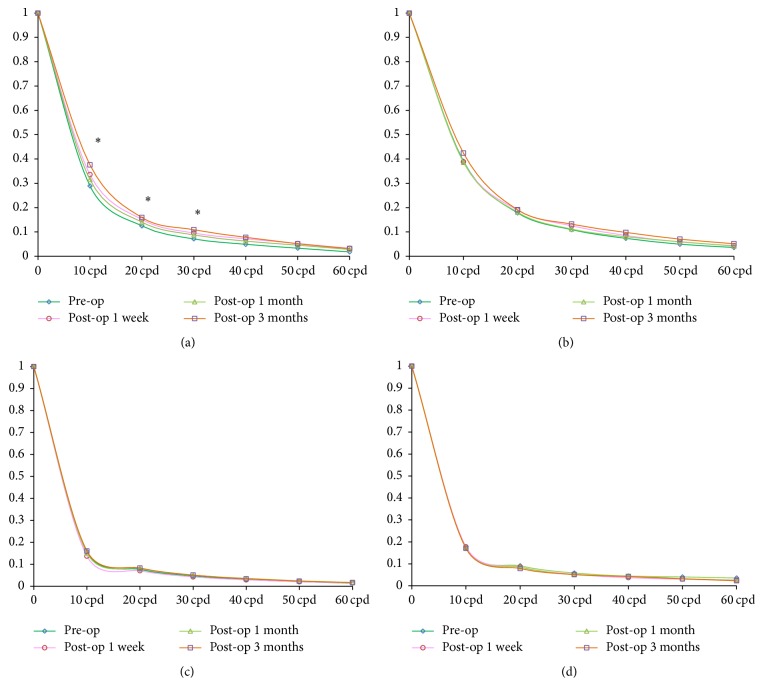
The changes of MTF values before and 1 week, 1 month, and 3 months after Femto-LASIK procedure. (a) The change of MTF curve at vertical meridian at 3 mm pupil diameters; (b) the change of MTF curve at horizontal meridian at 3 mm pupil diameters; (c) the change of MTF curve at vertical meridian at 6 mm pupil diameters; (d) the change of MTF curve at horizontal meridian at 6 mm pupil diameters. ^*∗*^Significant difference among different follow-up time.

There was significant increase in the MTF values of anterior corneal surface at vertical meridian in low and mediate frequency (10, 20, and 30 cpd) at 3 mm pupil diameter before and after Femto-LASIK procedure (*P* < 0.05 for all) 1 week, 1 month, and 3 months postoperatively. However, there was no significant difference in the MTF values at horizontal meridian from low to high frequency at 3 mm optical zone before and after Femto-LASIK procedure (*P* < 0.05 for all) (Figures 2(a) and 2(b)). And there was also no significant difference in the MTF values at both vertical and horizontal meridian from low to high frequency at 6 mm pupil diameter during the follow-up time (Figures 2(c) and 2(d)).

### 3.4. Changes in ΔMTF Values (Postoperative-Preoperative MTF Value) between SMILE and Femto-LASIK Procedures

**Figure 3:**
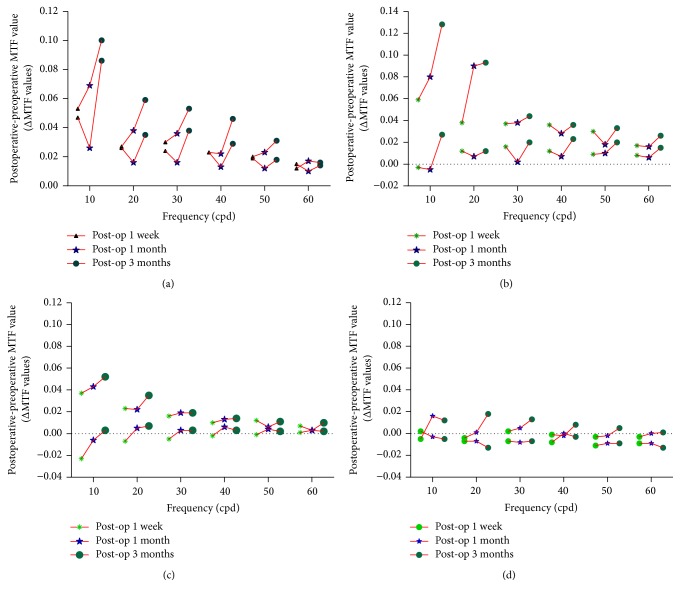
The changes in ΔMTF values (postoperative-preoperative MTF value) between SMILE and Femto-LASIK procedures at 1 week, 1 month, and 3 months postoperatively (up broken lines: Changes of MTF values at 1 week, 1 month, and 3 months after SMILE procedure at different spatial frequency; lower broken lines: changes of MTF values at 1 week, 1 month, and 3 months after Femto-LASIK procedure at different spatial frequency). (a) The change of MTF at vertical meridian at 3 mm pupil diameters; (b) the change of MTF at horizontal meridian at 3 mm pupil diameters; (c) the change of MTF at vertical meridian at 6 mm pupil diameters; (d) the change of MTF at horizontal meridian at 6 mm pupil diameters.

**Figure 4:**
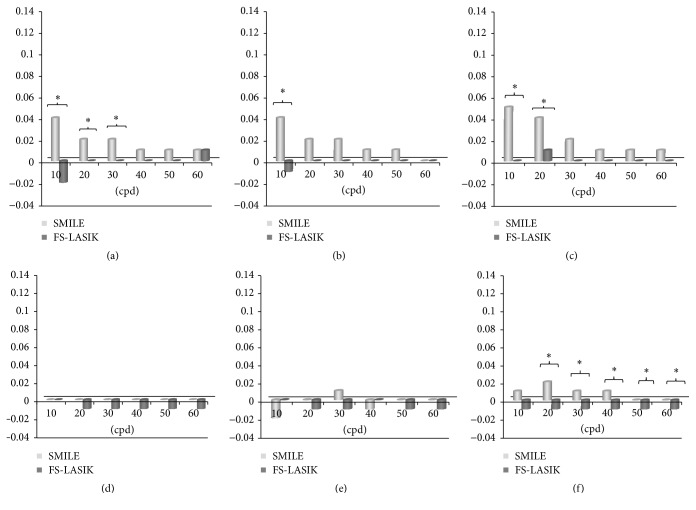
The changes in ΔMTF values at 6 mm pupil diameter (postoperative-preoperative MTF value) between SMILE and Femto-LASIK procedures at 1 week, 1 month, and 3 months postoperatively. (a) The change of ΔMTF at vertical meridian at 1 week postoperatively; (b) the change of ΔMTF at vertical meridian at 1 month postoperatively; (c) the change of ΔMTF at vertical meridian at 3 months postoperatively; (d) the change of ΔMTF at horizontal meridian at 1 week postoperatively; (e) the change of ΔMTF at horizontal meridian at 1 month postoperatively; (f) the change of ΔMTF at horizontal meridian at 3 months postoperatively. ^*∗*^Significant differences (*P* < 0.05) in ΔMTF between SMILE and Femto-LASIK group.

**Figure 5:**
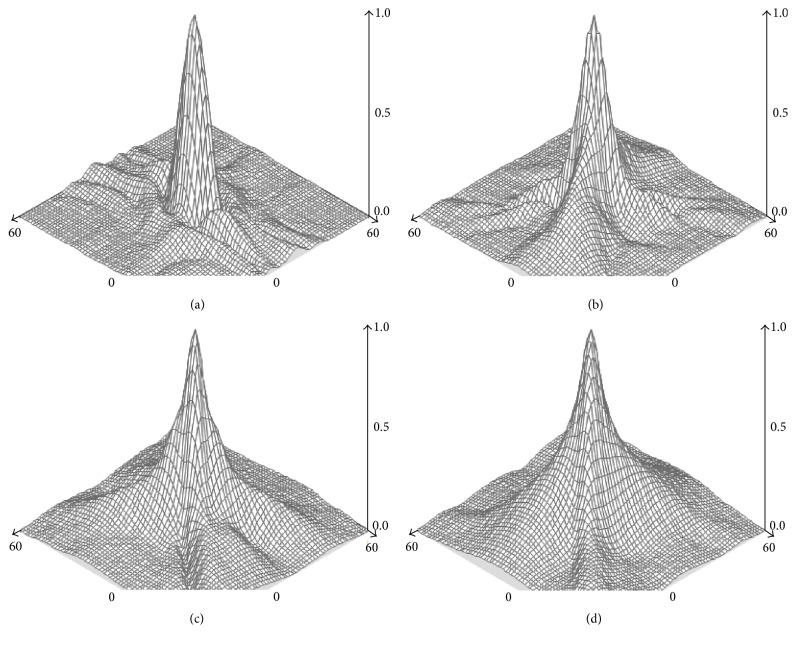
The three-dimensional images of MTF values ((a) before procedure; (b) 1 week after procedure; (c) 1 month after procedure; (d) 3 months after SMILE procedure).

As shown in Figure 3, it can be seen that the MTF values decrease following the increase of the frequency for both two groups. And the values in SMILE group were almost all higher than those in Femto-LASIK group. There was no significant difference in ΔMTF values from low to high frequency at vertical and horizontal meridian of 3 mm pupil diameter between SMILE and Femto-LASIK procedures at 1 week, 1 month, and 3 months (*P* > 0.05 for all) (Figures 3(a) and 3(b)). However, significant differences in ΔMTF values were shown at low frequency of vertical meridian at 6 mm pupil diameter during all the follow-up time (*P* < 0.05 for all) (Figures 4(a), 4(b), and 4(c)). This showed a significant difference from low to high frequency of horizontal meridian 3 months postoperatively (*P* < 0.05 for all) (Figure 4(f)). From the three-dimensional images of MTF values, it can be found that SMILE showed an improvement in optical quality (Figure 5).

### 3.5. Changes in ΔStrehl Ratio Values between SMILE and Femto-LASIK Procedures

**Figure 6:**
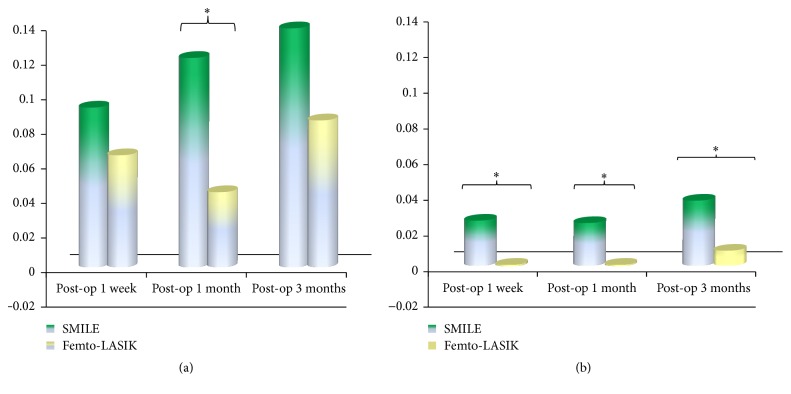
Changes in ΔStrehl ratio values between SMILE and Femto-LASIK procedures ((a) 3 mm pupil diameter, (b) 6 mm pupil diameter). ^*∗*^Significant differences (*P* < 0.05) in ΔStrehl ratio values between SMILE and Femto-LASIK procedures.

Significant differences in ΔStrehl ratio values of 6 mm pupil diameter between SMILE and Femto-LASIK procedures were shown between SMILE and Femto-LASIK procedures at 1 week, 1 month, and 3 months postoperatively (*P* = 0.038, 0.039, and 0.023, resp.). However, significant difference in ΔStrehl ratio values of 3 mm pupil diameter was only found at 1 month postoperatively (*P* < 0.05) between SMILE and Femto-LASIK procedures (Figure 6).

### 3.6. Changes in ΔRMS Values between SMILE and Femto-LASIK Procedures

Significant differences in ΔRMS values of 3 mm pupil diameter were found between SMILE and Femto-LASIK procedures at 1 month and 3 months postoperatively (*P* < 0.05). However, there was no significant difference in ΔRMS of 6 mm pupil diameter during the follow-up time (*P* > 0.05 for all) (Table 2).

## 4. Discussion

Small incision lenticule extraction (SMILE), as a novel technique of refractive correction, has been widely used in the correction of myopia and myopic astigmatism. The clinical outcomes of SMILE used to correct refractive error have been demonstrated generally [6–8]. Visual acuity and high-order aberration after SMILE procedure have also been studied [8]. However, to our knowledge, there are less studies on optical quality after SMILE procedure. This study was conducted to compare the MTF and PSF values changes between SMILE and Femto-LASIK surgery in order to evaluate the optical quality of the early stage postoperatively.

The result showed that the MTF curve of the corneal surface at vertical and horizontal meridian increases significantly from low to high frequency at 3 mm pupil diameter before and after SMILE procedure during the follow-up time. And it only showed significant increase at vertical meridian of low and high frequency (10, 20, and 30 cpd) at 3 mm pupil diameter after Femto-LASIK procedure. Some reasons may contribute to this difference in MTF values after SMILE and Femto-LASIK surgery. Firstly, MTF analyzes the image contrast as a function of frequency. The low frequency reflects the capability to identify an object's contour. The mediate frequency reflects the transfer capability of the objects' layers, and it can indicate the outcomes of visual acuity and contrast sensitivity. The high frequency of the curve reflects the transfer capability of the objects' details [9]. Regarding the visual outcomes, our results suggest that both two groups experienced highly effective myopia correction. Therefore, both groups showed significant improvement in low and high frequency at 3 mm pupil diameter during the follow-up time.

Secondly, the optical quality of human eyes can be affected mainly by defocus while the effect of high-order aberrations only takes 10%*~*20%. After surgery, the defocus was corrected. And the MTF values would be improved.

However, for Femto-LASIK group, there was no significant difference in MTF values of high frequency at 3 mm pupil diameter after surgery comparing to preoperative. Many previous studies have shown that small irregularities in the stromal surface, such as tissue bridges and interface debris, can lead to light scatter and elevated straylight values [10, 11]. Our previous study shows that straylight increased significantly in the early stages after Femto-LASIK [12] and there was more increase in straylight than those after SMILE [13]. Therefore, we speculate that increased straylight may affect the results of MTF after Femto-LASIK.

We also found that there was no significant difference in the MTF values at horizontal meridian from low to high frequency at 3 mm pupil diameter before and after Femto-LASIK procedure. That might be related to the direction of the flap. The pedicle of the flap is located on the nasal side. Thus, the transverse wound healing may decrease the MTF value. For the SMILE surgery, one study [14] has demonstrated that the early inflammatory and wound healing response were minimal. This might be another reason why the MTF improved after SMILE surgery.

Our previous study [15] found that average optical quality in those eyes after correction of sphere and cylinder was dependent on pupil size. Over a large range of spatial frequencies, the average ΔMTF in the 3 mm pupils diameter were almost identical between SMILE and Femto-LASIK eyes, while for 6 mm pupil diameter the MTF was much lower than those for the 3 mm pupil diameter across all spatial frequencies. It also can be seen that, with a 6 mm pupil diameter, SMILE was higher than that for Femto-LASIK at spatial frequencies less than 60 cpd during the follow-up time. The ΔMTF also shows that much more changes in low frequency at vertical meridian of 6 mm pupil diameter were found in SMILE group than Femto-LASIK group at 1 week, 1 month, and 3 months postoperatively. At horizontal meridian, significant differences were found in MTF values from low to high frequency at 3 months after surgery. The ΔStrehl ratio also shows a significant difference between SMILE and Femto-LASIK group at 1 month at 3 mm pupil diameter and at 1 week, 1 month, and 3 months postoperatively at 6 mm pupil diameter. Thus, from these, it can be seen that the optical quality of SMILE is better than that of Femto-LASIK, especially at lager pupil diameter.

Higher-order RMS and MTF were both used to evaluate image quality, although the exact relationship between the two metrics was dependent on the relationships between Zernike coefficients [15]. One of our previous studies showed that, for an equal increase of pupil size, not all Zernike polynomial coefficients induced equivalent increase of values. Coma-like aberrations had less increase following the pupil dilation. Spherical-like aberration and other higher-order aberrations also showed slight increase following the pupil dilation [16]. In this current study, significant differences in ΔRMS values of 3 mm pupil diameter were found between SMILE and Femto-LASIK procedures at 1 month and 3 months postoperatively. However, there were no significant differences in ΔRMS of 6 mm pupil diameter during the follow-up time. This means that, at small pupil diameter, SMILE induces less change in RMS than Femto-LASIK surgery. However, at big pupil diameter, maybe the effect of pupil size on high-order aberration hides the effect of surgery itself. At the early stage after the surgery such as at 1 week, corneal wound healing maybe induces some aberration in both groups. However, it might mitigate quickly after SMILE compared to the Femto-LASIK surgery.

A study showed [17] that MTF value of cornea may relate to the tear film stability. SMILE procedure is flapless. Instead of about 300° side cut, only a small 50° side-cut incision is made. For Femto-LASIK procedure, the 8.0 mm diameter flap is created by femtosecond laser. More corneal nerves are cut during the flap creating. Therefore, patients are more likely to suffer dry eye after Femto-LASIK than SMILE procedure. It was presumed that the MTF values after Femto-LASIK are more likely affected by tear film quality than those after SMILE surgery. A study shows that ocular forward light scattering from the anterior cornea was greater in dry eyes than in normal eyes [18]. Thus, in this study, we can find that the ΔStrehl ratio in Femto-LASIK shows less change than in SMILE group during the follow-up time.

In conclusion, SMILE shows a great improvement in optical quality both under small and lager pupil diameter at early stage postoperatively. Femto-LASIK has shown improvement in optical quality at small pupil diameter. SMILE shows better optical quality than Femto-LASIK at lager pupil diameter. It means that better night vision might be shown after SMILE procedure. However, further investigations on the optical quality may be needed due to the complex nature of visual system.

## Figures and Tables

**Table 1:** Preoperative patients characteristics.

Group	Mean ± SD^a^		*F*	*P*
	SMILE	FS-LASIK		
Age, years	22.7 ± 5.6	23.30 ± 4.14	0.463	0.499
SD, D	−5.13 ± 1.25	−5.90 ± 1.85	8.605	0.058
MRSE, D	−5.55 ± 1.23	−5.84 ± 1.90	9.787	0.055
CCT, *μ*m	551.78 ± 24.10	540.48 ± 31.55	3.076	0.089
Mean K, D	43.32 ± 1.24	41.96 ± 1.26	0.347	0.547

ANOVA test.

SD^a^: standard deviation; SD: spherical degree; MRSE: manifest refraction spherical equivalent; K: Pentacam keratometry; CCT: central corneal thickness.

**Table 2:** ΔRMS at different pupil diameter between SMILE and Femto-LASIK procedure.

Pupil diameter (*μ*m)	Time	Group (mean ± SD)		*t*	*P*
		SMILE	Femto-LASIK		
3 mm	Post-op 1 week	0.070 ± 0.208	0.010 ± −0.255	−1.886	0.062
	Post-op 1 month	0.103 ± −0.182	0.053 ± 0.473	−2.561	0.012^*∗*^
	Post-op 3 month	0.119 ± 0.172	0.054 ± 0.131	−2.325	0.022^*∗*^
6 mm	Post-op 1 week	0.123 ± 1.005	0.459 ± 1.507	−1.433	0.152
	Post-op 1 month	−0.085 ± 0.693	0.364 ± 1.389	−1.628	0.089
	Post-op 3 month	−0.106 ± 0.128	0.593 ± 0.770	−1.875	0.063

Mann–Whitney *U* nonparametric test.

SD: standard deviation; Pre-op: preoperation; Postop: postoperative.

^*∗*^Significant differences (*P* < 0.05) in ΔRMS between SMILE and Femto-LASIK procedure.
